# A novel pilus-associated gene cluster is implicated in *Streptococcus agalactiae* virulence

**DOI:** 10.1186/s13567-025-01567-z

**Published:** 2025-07-03

**Authors:** Wanyou Xu, Qiangsheng Lu, Meijuan Tian, Lvfeng Yuan, Yangming Song, Qiaoying Zeng, Jie Peng

**Affiliations:** 1 College of Veterinary Medicine, Gansu Agricultural University, Gansu, China; 2 Lanzhou Veterinary Research Institute, Chinese Academy of Agricultural Sciences, Lanzhou, China; 3 State Key Laboratory of Veterinary Etiological Biology, College of Veterinary Medicine, Lanzhou University, Lanzhou, China

**Keywords:** *Streptococcusagalactiae*, adhesion, pilus-related genes, pathogenicity

## Abstract

*Streptococcus agalactiae* (*S. agalactiae*), also known as Group B Streptococcus (GBS), is an important zoonotic pathogen. It is an important pathogen that causes bovine mastitis and can also lead to severe infections in newborns, pregnant women, and individuals with a weakened immune system. Adhesion to host cells is the first critical step in *S. agalactiae* infection, making the identification of key adhesion-related genes essential for understanding the pathogenic mechanisms of GBS. In this study, we used the Tn transposon system to construct a mutant library of *S. agalactiae* A909 and employed the mouse mammary epithelial cell line EPH4-Ev as a model to screen and validate adhesion-related genes. Our results revealed a gene cluster containing BP, AP1, and SrtC. While these genes did not affect the basic biological characteristics of the bacteria, they significantly influenced the adhesion rate to cells and the virulence of A909 in mice. Further transcriptomic analysis revealed that the *Tnfsf15* gene plays a critical role in the regulation of A909 strain pathogenicity mediated by the BP, AP1, and SrtC gene clusters during host infection.

**Supplementary Information:**

The online version contains supplementary material available at 10.1186/s13567-025-01567-z.

## Introduction

*Streptococcus agalactiae*, a Gram-positive bacterium, is widely distributed in both human and animal environments worldwide and has become a significant pathogen in the field of public health [1, 2]. According to statistics, cases of bovine mastitis caused by *S. agalactiae* account for up to 9% of dairy cattle farming cases, seriously restricting the healthy development of the industry [3–5]. Bovine mastitis not only damages the mammary gland tissue of dairy cows and reduces milk production but also decreases key nutrients such as fat and protein in milk, severely affecting the quality and commercial value of dairy products [3, 6]. In addition, *S. agalactiae* is zoonotic, and if humans consume contaminated milk, the risk of infection increases significantly, posing a potential threat to public health [7, 8].

Research has shown that once *S. agalactiae* enters tissues, it utilizes its virulence factors to interact with host cells, facilitating adhesion and invasion, which lays the foundation for subsequent colonization, reproduction, and pathogenesis [9, 10]. Therefore, screening for novel virulence genes associated with the adhesion and invasion of *S. agalactiae* can contribute to a more in-depth exploration of its pathogenic mechanism, facilitate the identification of key functional molecules involved in the infection process, and fill and refine the existing gaps in our understanding of the virulence genes of A909. Transposon mutagenesis libraries have been widely applied, especially in gene function research. The development of second-generation sequencing technologies has enabled more accurate and efficient analysis of microbial genomes, and transposon-related data have been integrated into various databases. The use of transposon mutagenesis libraries to screen for virulence genes offers significant advantages. The transposon system allows for the rapid construction of a large number of mutants, and because transposons are inserted randomly into the genome, they can act on various gene regions without bias, increasing the likelihood of discovering novel functional genes. The inserted transposons are stably inherited, facilitating long-term research and analysis. Additionally, screening mutants on the basis of phenotypic traits is intuitive and convenient, enabling the rapid identification of genes associated with specific characteristics, thereby reducing research complexity and improving screening accuracy [11, 12].

In this study, a transposon mutant library of A909 was constructed using the Tn transposon system, and a virulence gene cluster associated with the adhesion and invasion of mammary epithelial cells by *S. agalactiae* was identified through high-throughput sequencing. This gene cluster has not been previously reported in A909. Through comparative genomic analysis, the three genes within the cluster were designated BP, AP1, and SrtC. Experimental validation further confirmed that this gene cluster is closely related to the virulence of A909. In terms of the functional mechanism, this newly discovered gene cluster encompasses only the key components involved in the pathogenic effect of the pili of *S. agalactiae*. Among them, as a pilin backbone protein-encoding gene, BP encodes the core structural unit of the pili. It mediates the adhesion of bacteria to target cells such as mammary epithelial cells and nerve cells by specifically recognizing glycoproteins and glycolipid receptors on the surface of host cells [13–15]. As an auxiliary protein-encoding gene, AP1 plays a crucial role in the assembly process of pili. It participates in the transportation, positioning, and orderly connection of pilin subunits to ensure the correct folding and extension of the pili [16, 17]. While SrtC is a sortase gene, the sortase C enzyme encoded by it can recognize the LPXTG motif at the C-terminus of the pilin protein. Through a hydrolysis-transpeptidation reaction, the pilin protein is covalently anchored to the peptidoglycan of the cell wall, enabling the pili to be stably displayed on the surface of the bacteria [18, 19]. To further elucidate the functional mechanism of the BP, AP1, and SrtC gene clusters in the development of mastitis caused by A909, this study analysed the impact of these genes on transcriptomic expression in EPH4-Ev cells. Preliminary findings suggest that *Tnfsf15* may serve as a key host molecule regulated by this gene cluster to mediate inflammation and tissue damage during mastitis. As a core component of the immunomodulatory network, *Tnfsf15* is involved in regulating various inflammation-related signalling pathways. For example, it activates the NF-κB signalling pathway, precisely modulating the chemotaxis, activation, and migration of inflammatory cells. This mechanism promotes the recruitment of immune cells such as neutrophils and macrophages to infection sites, thereby enhancing the innate immune response [20, 21]. In addition, *Tnfsf15* also regulates the expression and secretion of multiple cytokines, including interleukin-6 (IL-6) and interferon-gamma (IFN-γ), which play key roles in maintaining the dynamic balance between tissue repair and damage [22, 23]. Mastitis caused by *S. agalactiae* infection of bovine mammary tissue is characterized by typical pathological features such as inflammatory cell infiltration and damage to mammary epithelial cells. These pathological phenotypes closely resemble the immunopathological processes mediated by *Tnfsf15*. Therefore, on the basis of the consistency between pathological phenotypes, transcriptomic analysis, and validation results, it is speculated that *Tnfsf15* is a key host molecule regulated by the *S. agalactiae* BP, AP1, and SrtC gene clusters during host infection.

In this study, the key gene clusters BP, AP1, and SrtC, associated with the adhesion and invasion capabilities of A909, were successfully identified through a transposon mutant library. This gene cluster is closely related to bacterial virulence. Additionally, the potential mechanism by which this cluster contributes to the development of mastitis was preliminarily explored. These findings provide a solid experimental foundation for further elucidating the pathogenic mechanisms of *S. agalactiae*.

## Materials and methods

### Strains and plasmids

The *S. agalactiae* strain A909 (A909) and the plasmids used for gene knockout (pMar4s) were stored in our laboratory. In this study, A909 was cultured in Tryptic Soy Broth (TSB) medium, while the pMar4s, pSET4s and pAC-GFP-*Tnfsf15* plasmids were stored in *Escherichia coli DH5α* and cultured in LB medium.

### Animals and cells

Pregnant Kunming mice were purchased from the Lanzhou Veterinary Research Institute, Chinese Academy of Agricultural Sciences. All animal experiments were conducted in accordance with the guidelines of the Gansu Provincial Department of Science and Technology (SYXK20200010), and the protocol was approved by the Standing Committee on Animal Protection of the Lanzhou Veterinary Research Institute. Mouse mammary epithelial cells (EPH4-Ev) and bovine mammary epithelial cells (MAC-T), purchased from ATCC, were stored in our laboratory. These cells were cultured in Dulbecco’s modified Eagle’s medium (DMEM) supplemented with 10% foetal bovine serum.

### Pathogenicity test of S. agalactiae A909

Postpartum days 7–10, the mice were randomly divided into two groups, the negative control group and the A909 (wild-type, WT) infection group, with 3 mice in each group. WT was injected via the mammary duct, whereas the control group received an equal volume of PBS. Forty-eight hours after infection, the mice were euthanized humanely, and mammary tissues were collected. One side of the mammary tissue sample was fixed in 4% formaldehyde for tissue sectioning and H&E staining to observe histopathological changes; the other side was used to assess the bacterial load and measure the expression levels of IL-1α, IL-6, IL-10, and tumor necrosis factor-α (TNF-α). The relative quantification of inflammatory cytokine expression levels was performed using the 2^−∆∆Ct^ method. The primer sequences are shown in Table 1.

**Table 1:** **Primer sequences for quantitative real-time polymerase chain reaction**

Primer name	Primer sequence
il1a-qF	ACCCAGATCAGCACCTTACACC
il1a-qR	CTCCTCCCGACGAGTAGGCAT
il6-qF	TCTGGAGCCCACCAAGAACGA
il6-qR	ACATGTGTAATTAAGCCTCCGAC
il10-qF	CTGCACCCACTTCCCAGTCG
il10-qR	ACTGGATCATTTCCGATAAGGC
tnf-qF	AGCACAGAAAGCATGATCCG
tnf-qR	AGCTGCTCCTCCACTTGGT
gapdh-qF	GCGACTTCAACAGCAACTCCC
gapdh-qR	CACCCTGTTGCTGTAGCCGTA

### Construction and verification of the whole-genome transposon mutant library of S. agalactiae A909

The pMar4s plasmid, constructed by Liu et al. [24], was used to create the whole-genome transposon mutant library of A909. The pMar4s plasmid was electroporated into competent A909 cells. Immediately after electroporation, the bacteria were suspended in recovery solution (a TSB solution containing 0.2% yeast extract and 30 nM sucrose) and cultured with shaking at 28 °C and 180 rpm for 3 h. The transformed bacteria were then spread on a TSB plate containing spectinomycin (Spc, 100 μg/mL) and cultured overnight at 28 °C to ensure that the pMar4s plasmid replicated as an independent plasmid.

More than 100 colonies were scraped from each Petri dish into a test tube, resulting in a total of 24 tubes. After dilution, the bacteria were spread onto Spc plates, kanamycin (Kan, 100 μg/mL) plates, and regular TSB plates and cultured at 37 °C for 24 h. The integration frequency for each test tube was calculated as the ratio of the number of colonies on the Spc plate to the total number of colonies on the TSB plate. Test tubes with an integration frequency of less than 0.1% were selected as insertion mutants. The diluted selective insertion mutants from each test tube were spread onto Kan and Spc plates (expected concentration: 8 × 10^5^–1 × 10^6^) to verify the integration frequency. Colonies from each Kan plate were then scraped to construct a gene mutant library, covering approximately 100 times the entire genome of A909 (bacterial count > 2.3 × 10^6^ CFU). Ten transposon mutant strains were randomly selected. The insertion of the transposon TnYLB-1 gene fragment was detected by PCR, and the randomness of the transposon insertion site was further confirmed using reverse PCR.

### Screening and analysis of virulence genes related to the adhesion and invasion of S. agalactiae A909

EPH4-Ev cells were cultured in DMEM supplemented with 10% foetal bovine serum and seeded evenly into 12-well plates to form a monolayer. Twenty microlitres of the mutant library bacterial suspension was spread onto a TSB plate containing Kan (100 μg/mL) and incubated overnight at 37 °C. The plate was then rinsed with 1.5 mL of TSB liquid medium, and the rinse solution was collected, centrifuged, and washed. The bacterial cells were resuspended in serum-free DMEM and adjusted to an OD_600_ of 1.0. After a tenfold dilution, 500 μL of the bacterial suspension was inoculated into 12-well plate.

After incubation for 2–4–6 h, the wells were rinsed twice with serum-free DMEM. For the 2-h and 4-h treatment groups, 500 μL of sterile water was added directly to lyse the cells, and the lysate was collected. For the 6-h treatment group, 500 μL of DMEM (containing penicillin‒streptomycin, 100 μg/mL) was added and incubated for an additional 1 h. Then, the wells were rinsed twice with serum-free DMEM, the rinse solution was discarded, 500 μL of sterile water was added to lyse the cells, and the lysate was collected.

The lysates were sent to Beijing Novogene Technology Co., Ltd., for bacterial genome sequencing. The raw data were filtered using Trim Galore (version 0.6.7) and aligned to the A909 reference genome (GCF_000012705) and the transposon sequence using Bowtie2 (version 2.5.1). Transposon insertion sites and chimeric sequences were identified using Python3, and statistical analyses were conducted with the scipy package. Data visualization was performed using GraphPad and Python packages, including matplotlib, seaborn, and SnapGene. Each group included three biological replicates, with the negative control consisting of an equal volume of diluted mutant library.

### Effects of pilus-related genes of S. agalactiae A909 on bacterial growth performance

The BP, AP1, and SrtC gene deletion mutants of A909 were constructed using the homologous recombination method. After subculture and screening, suspected colonies were picked for PCR identification and sequencing. After verification, the mutants were named ΔBP, ΔAP1, and ΔSrtC. The successfully constructed gene deletion mutants were transferred to TSB liquid media at a ratio of 1:100 (v/v) and serially passaged for 50 generations. The genetic stability of the deletion mutants was detected by PCR.

ΔBP, ΔAP1, and ΔSrtC were cultured to the logarithmic growth phase (OD_600_ = 0.6–0.8), inoculated into 100 mL of TSB liquid medium without antibiotics at a ratio of 1:100 (v/v), and cultured at 37 °C and 180 rpm. The OD_600_ was measured every 1 h for 12 h. A bacterial growth curve was drawn with the culture time as the abscissa and the OD_600_ as the ordinate. Each group had three parallel controls.

The ΔBP, ΔAP1, and ΔSrtC strains were centrifuged in the logarithmic growth phase, and the precipitates were collected. After rinsing with PBS, the samples were suspended and fixed with 3% glutaraldehyde. After fixation with 1% osmium tetroxide for 1–2 h, the samples were washed with ultrapure water and divided into two parts. One part was dehydrated with an alcohol gradient, dropped onto a silicon wafer, attached to the sample stage with conductive glue, and sprayed with gold. These samples were observed and photographed using a scanning electron microscope (SEM, JSM-IT700HR). The other part was dehydrated with an acetone gradient, infiltrated and embedded with a dehydrating agent and embedding agent. Ultrathin sections were prepared, picked on a copper grid, stained, and observed and photographed by transmission electron microscopy (TEM, JEM-1400FLASH).

### Transcriptomic analysis of the effects of pilus-related genes on the function of S. agalactiae A909

The ∆BP, ∆AP1, ∆SrtC, and WT A909 strains were cultured to the logarithmic growth phase, and the OD_600_ was adjusted to 1.0. After tenfold dilution, 2 mL of the diluted bacterial suspension was inoculated into a 6-well cell culture plate seeded with EPH4-Ev cells and incubated for 2 h. The wells were then rinsed twice with serum-free DMEM, and the rinse solution was discarded. Next, 1 mL of TRIzol reagent was added to lyse the cells. The lysate was collected and sent to Beijing Novogene Technology Co., Ltd., for transcriptome sequencing and analysis. Each group had three parallel controls, with the negative control receiving only serum-free DMEM. The results of the transcriptome analysis were validated using quantitative qRT-PCR method. GAPDH was used as the reference gene, and the 2^−ΔΔCt^ method was applied to calculate the relative expression levels of each gene. The primer sequences are shown in Additional file 1.

### Knockdown and overexpression of the Tnfsf15 gene in EPH4-Ev cells

The small interfering RNA (siRNA) of *Tnfsf15* was purchased from Genomeditech Biotechnology Co., Ltd. (Shanghai, China). The sequences of the small interfering RNAs used were as follows: 5′-AAAUCAGCUCUCUGCUCUAtt-3′ and 5′-UAGAGCAGAGAGAGCUGAUUUtt-3′. The overexpression plasmid of *Tnfsf15* was constructed by synthesizing the *Tnfsf15* gene and inserting it into the multiple cloning site of the pAC-GFP plasmid and was named pAC-GFP-*Tnfsf15*. Lipofectamine 3000 (Thermo Fisher Scientific) was used to transfect the siRNA and pAC-GFP-*Tnfsf15* into EPH4-Ev cells. Forty-eight hours after transfection, the expression level of *Tnfsf15* mRNA was quantified by real-time PCR to evaluate the efficiency of knockdown or overexpression.

### Statistical analysis

Three technical replicates were performed for each independent experiment. Statistical analysis was conducted using one-way analysis of variance (ANOVA) and Student’s *t* test (two-tailed). The data were analysed using GraphPad Prism 9.5.0 statistical software (Chicago, Illinois, USA). The quantitative data are presented as the means ± standard deviations, with *P* < 0.05 considered statistically significant.

## Results

### Pathogenicity of S. agalactiae A909

**Figure 1:**
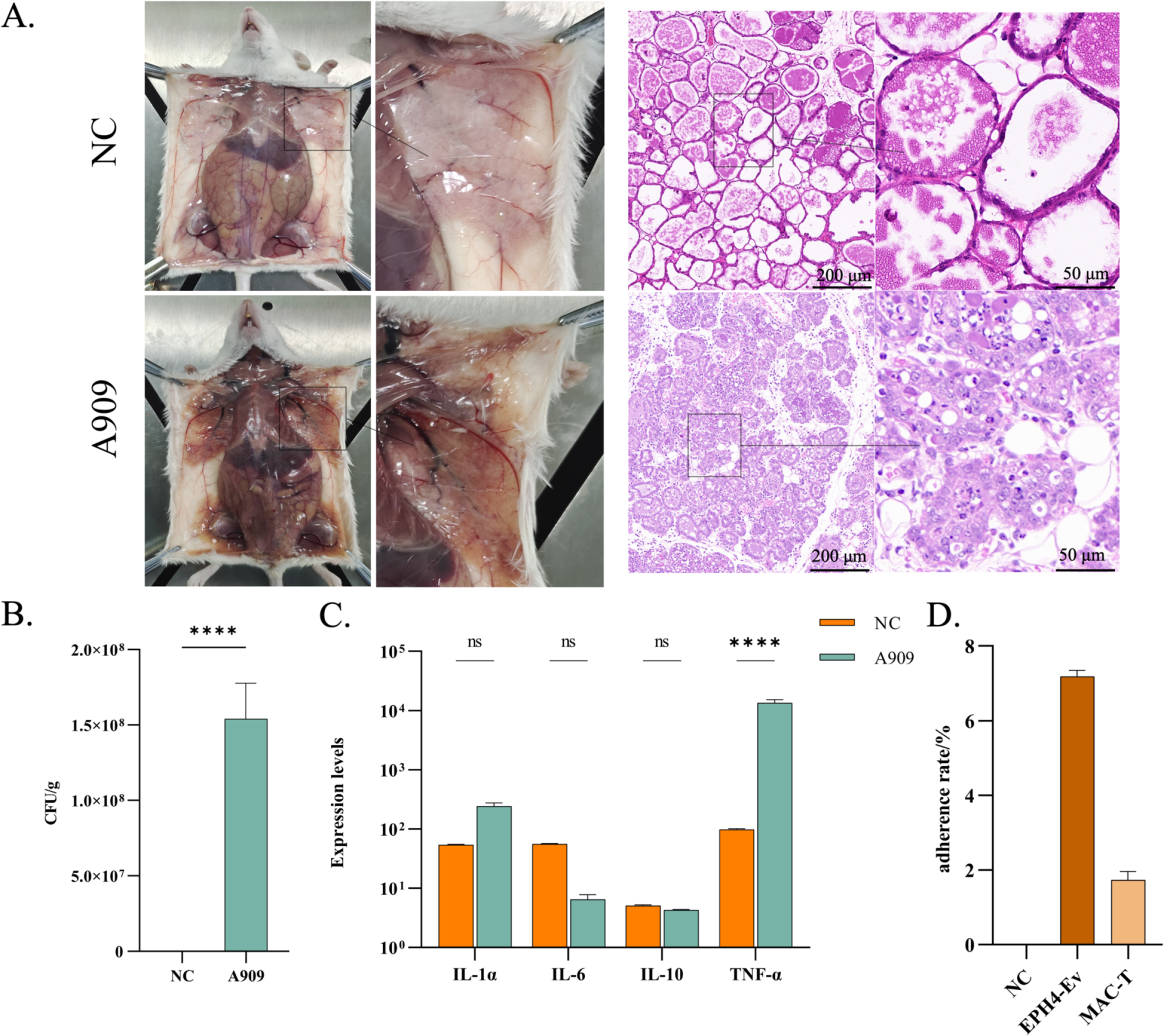
***S. agalactiae*** **A909 has strong pathogenicity.** **A** Histopathological examination of the mammary gland. Columns 1 and 2 show the gross pathological changes in the mouse mammary gland tissues; columns 3 and 4 show the microscopic observation results of the paraffin sections of the mouse mammary gland after H&E staining. **B** Results of the determination of the bacterial load in mouse mammary gland tissue. CFU: Colony forming unit. The data are presented as the mean ± standard deviation, ****P < 0.0001; Student’s *t* test. **C** Detection of the expression levels of inflammatory factors in mammary gland tissue by qPCR. The data are presented as the mean ± standard deviation, ****P < 0.0001; Student’s *t* test. IL: Interleukin, ns: Not significantly different, TNF: Tumour necrosis factor. **D** Determination of the adhesion ability of A909 cells. The data are presented as mean ± standard deviation.

We initially evaluated the ability of A909 to induce mastitis through both murine animal models and in vitro cell-based assays. In a mouse model, lactating mice infected with A909 displayed decreased responsiveness and pronounced erythema and swelling of the mammary glands. Upon necropsy, the mammary glands exhibited dark yellow colouration with evident hemorrhaging. In contrast, negative control mice remained active, and their mammary glands appeared milky white, were filled with abundant milk, and were free of pathological changes. Histopathological examination using H&E staining revealed that mammary gland tissue in the negative control group maintained an intact architecture, with clearly defined acinar boundaries and milk-filled alveoli. In the infected group, however, the acinar boundaries were indistinct, the acini were infiltrated with large numbers of neutrophils, and there was evidence of epithelial desquamation and necrosis (Figure 1A). Quantitative analysis of bacterial loads in mammary tissue revealed a markedly greater bacterial burden in the infected group (Figure 1B), indicating that A909 can effectively colonize mammary tissue and establish infection. As inflammatory cytokines are key indicators of tissue inflammatory responses, we further assessed the levels of four inflammatory factors in mammary tissue by qPCR. The results revealed significant upregulation of TNF-α expression in the infected group compared with the control group (Figure 1C), providing additional evidence that A909 induces a robust inflammatory response in murine mammary tissue and has strong pathogenic potential.

Furthermore, the adhesion abilities of A909 cells were examined in EPH4-Ev and MAC-T-cell lines. The adhesion rate of EPH4-Ev cells was 7.19 ± 0.16%, whereas that of MAC-T cells was 1.73 ± 0.23%. These results indicate that A909 is capable of adhering to and invading both murine and bovine mammary epithelial cells (Figure 1D). Collectively, these findings demonstrate that A909 can trigger mastitis by adhering to and invading mammary epithelial cells.

### Construction and validation of the whole-genome transposon mutant library of A909

**Figure 2:**
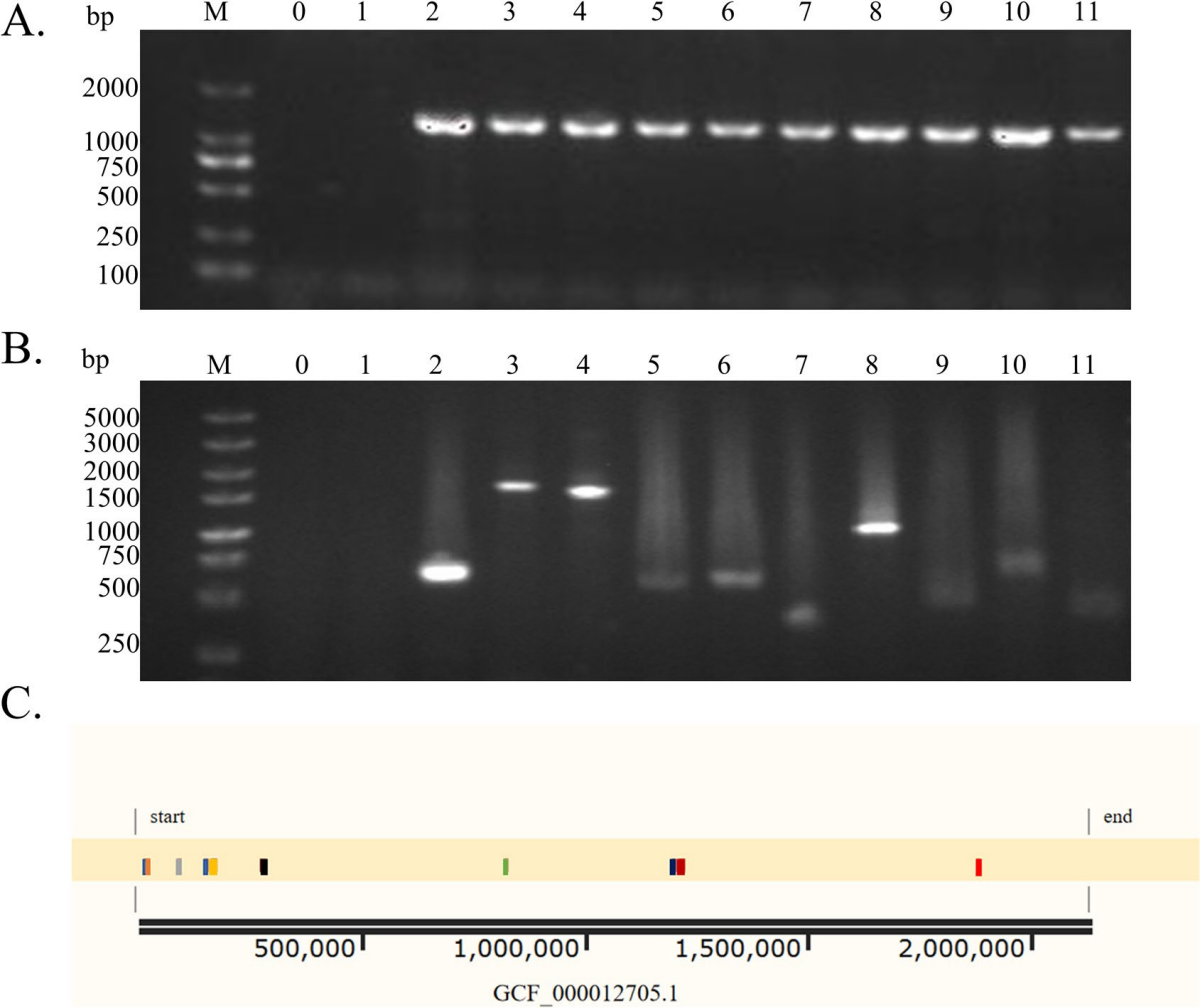
**Verification of transposon insertion and randomness in the mutant library.** **A** Electrophoresis diagram of PCR detection of the TnYLB-1 fragment in the genome of the A909 mutant strain. **B** Electrophoretogram of reverse PCR detection of flanking sequences of transposon insertion sites in the genome of the A909 mutant strain. (M: DNA marker; 0: negative control; 1: genome of WT A909; 2—11: genomes of 10 randomly selected mutant strains). **C** Alignment result diagram of the gene sequence at the transposon insertion site of the mutant strain with the sequence of the reference genome (GCF_000012705) of A909. (Squares of different colors represent genes at different insertion sites).

The pMar4S plasmid was electroporated into competent A909 cells. At 37 °C, the plasmid-borne transposon TnYLB-1 was induced to integrate into the genome of A909*.* The successfully transposed mutant strains presented kanamycin resistance but lacked resistance to spectinomycin. The mutant library obtained through resistance screening contains more than 2.3 × 10^6^ mutant strains. Compared with the 2173 genes in the genome of A909, the number of genes is large enough, and the generated mutation combinations have a high probability of covering all the gene loci. Ten random mutant strains were selected from the constructed library, and PCR was performed to check for the presence of the transposon TnYLB-1 within the mutant genomes. The results revealed that fragments of the same size as TnYLB-1 from the pMar4S plasmid were detected in all the selected mutants, whereas no fragments were detected in WT A909 (Figure 2A). These findings indicate that the transposon mutant strains isolated via resistance screening all contained TnYLB-1, achieving 100% coverage. The results of reverse PCR and sequencing alignment of the gel recovery products revealed that the transposons of the 10 mutant strains were all single insertions and that the insertion sites were different (Figures 2B and C). This mutant library can be used for further screening.

### Screening of genes involved in the adhesion and invasion of S. agalactiae A909 to EPH4-Ev cells

**Figure 3:**
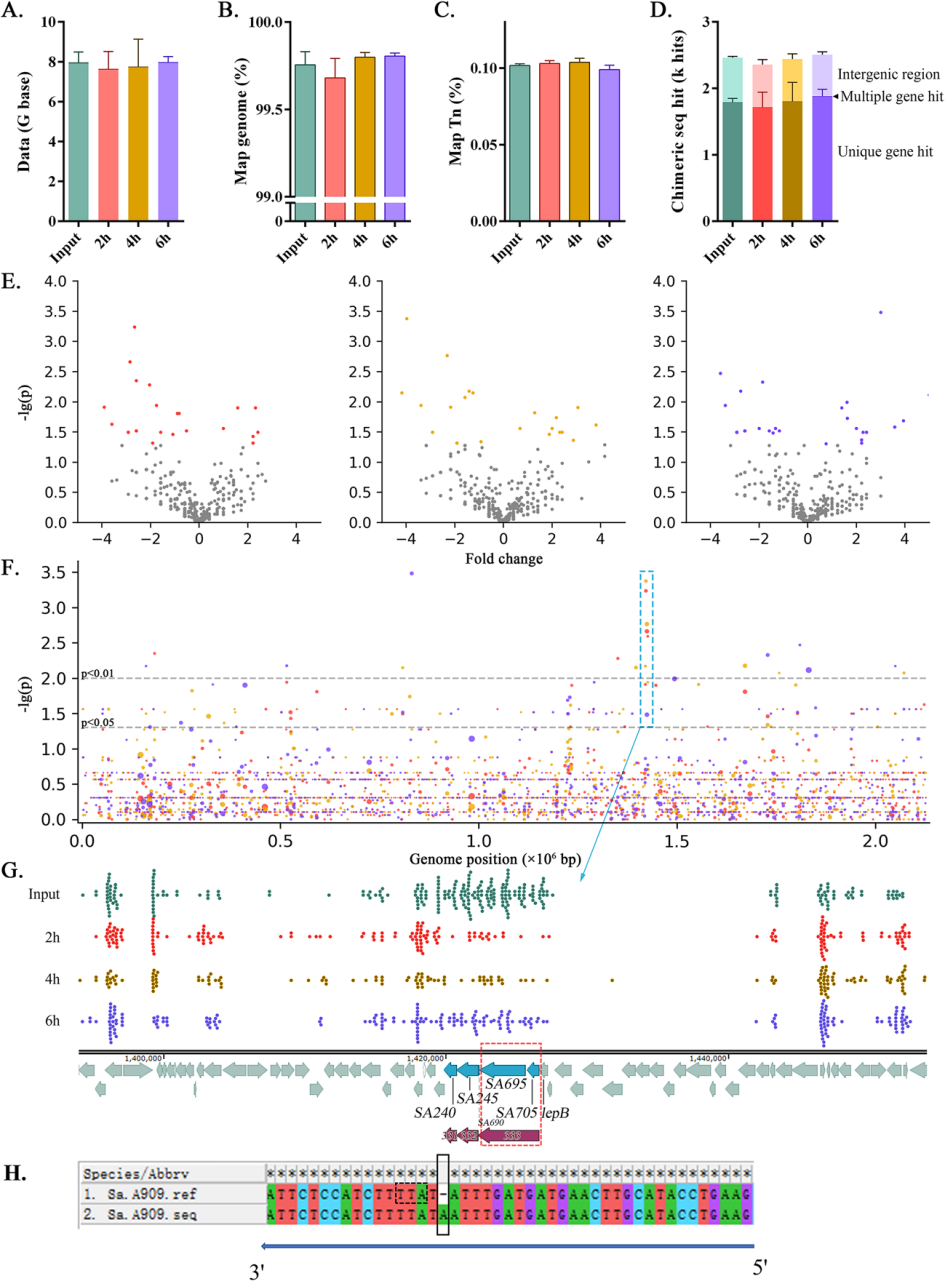
**Screening of adhesion-related genes in the *S. agalactiae *mutant library.** **A** Sequencing data volume and filtered data volume of the mutant library and adhesion test samples. The amount of data removed by filtering is shown as the dark part at the top of the column chart. **B** Column chart of the alignment rates of the data to the *S. agalactiae* reference genome. **C** Column chart of the alignment rate of the data to the transposon. **D** Column chart of the position types of the transposon insertion sites. **E** Volcano plot of the intergroup differences in the number of gene insertion mutations. From left to right are the intergroup comparisons of the 2 h, 4 h, and 6 h groups with the control group. The coloured dots indicate significant differences between genomes (*P* ≤ 0.05). **F** Scatter plot of the genomic position–intergroup difference *P* value of genes. The abscissa is the genomic position, and the ordinate is the intergroup difference *P* value. Red, yellow, and blue represent the intergroup comparisons of the 2 h, 4 h, and 6 h groups with the control group, respectively. The area of the dots is positively correlated with the number of detected chimeric sequences. **G** The upper part shows the distribution map of the mutant insertion sites within the 1.42 Gb amplification interval of the genome. The lower part shows the annotation information of the reference genome in this interval. Among them, SAK_RS07240 is marked as 331 in this paper, SAK_RS07245 is marked as 332, and SAK_RS11690, SAK_RS11695, and SAK_RS11705 are combined according to the sequencing results of this study and marked as 333 in this paper. **H** Comparison of the reference sequence (Sa.A909.ref) and the actual sequencing sequence (Sa.A909.seq) of *S. agalactiae* A909. An insertion mutation occurred in the region marked by the solid-line box in Sa.A909.seq. The base sequence corresponding to the dashed-line box in Sa.A909.ref forms a stop codon UAA on the mRNA after transcription, and this stop codon can cause the translation process to terminate prematurely.

Three biological replicates were performed for both the mutant library and the adhesion and invasion treatment groups, and bacterial genome sequencing was conducted for each group. The amount of sequencing data for each sample was approximately 8 Gb (Figure 3A). After adapters and low-quality sequences were filtered out, the sequencing yield was approximately 99.6%, with no significant differences between groups (Figure 3A). The data alignment rate to the *S. agalactiae* reference genome was approximately 99.8%, with no significant differences between groups (Figure 3B). The alignment rate with the transposon sequence was approximately 0.1%, with no significant differences between groups (Figure 3C). The ratio of the reference genome alignment to the transposon alignment was consistent with the ratio of sequence lengths, as expected. Upon identification, the chimeric sequence of the *S. agalactiae* genome and transposon was approximately 2.4 k per sample. Approximately 74% of the insertion sites were located within a single gene, approximately 26% were in intergenic regions, and approximately 0.1% were in gene overlap regions (Figure 3D). The number of insertion mutations in each gene was quantified, and intergroup difference analysis was performed (Figure 3E). The genomic position and intergroup difference *P* values for each gene are shown in Figure 3F. A distinct gene cluster was identified at the 1.42 Gb position of the genome (marked with a dashed box). Upon zooming in, the distribution map of the insertion sites in the mutant strains for each group is shown (Figure 3G). The intergroup differences in the number of mutants related to the genes SAK_RS07240, SAK_RS07245, SAK_RS11695, and SAK_RS11705 were highly significant. Additionally, a smaller gene, SAK_RS11690, located between SAK_RS07245 and SAK_RS11695, is not depicted in the figure.

By comparing the reference sequence (*Sa*. A909.ref) of A909 with the actual sequencing sequence (*Sa*. A909.seq), in the reference genome, there is a stop codon UAA at the corresponding position of the SAK_RS11705 gene (marked in the dashed box in Figure 3H), which means that the translation terminates at this position, and the subsequent major part is classified as another gene, SAK_RS11695 (marked in the dashed box in Figure 3G). However, the sequence obtained after actual sequencing and assembly shows that the entire region is a continuous gene 333 (marked in the dashed box in Figure 3G).

Sequence alignment via NCBI revealed that the protein encoded by SAK_RS07240 (331) has 100% homology with the class C sortase (SrtC) of group C *Streptococcus*, the protein encoded by SAK_RS07245 (332) has 99.80% homology with the backbone protein (BP) of *S. agalactiae* PI-2b, and the protein encoded by 333 has 100% homology with the ancillary protein 1 (AP1) of *Streptococcus pneumoniae*. Therefore, in this work, SAK_RS07240 (331) is referred to as SrtC, SAK_RS07245 (332) is labelled BP, and since SAK_RS11690 (not shown), SAK_RS11695, and SAK_RS11705 are fusion-expressed, they are collectively referred to as AP1.

### Effects of different genes encoding pilus-related proteins on the biological characteristics of S. agalactiae A909

**Figure 4:**
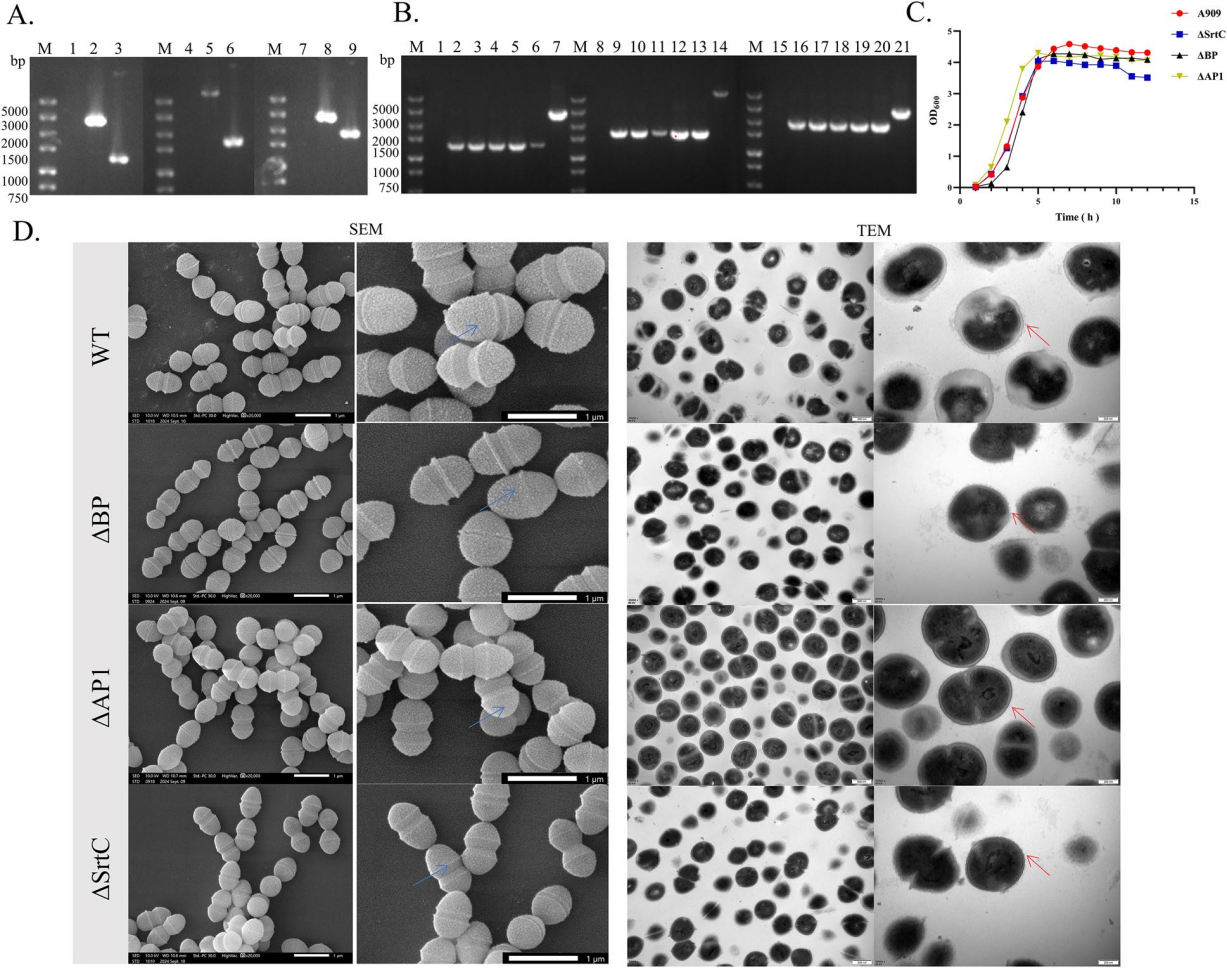
**Influence of pilus-related Genes on the Biological Characteristics of *S. agalactiae *A909.** **A** Electrophoresis diagram of the PCR identification of the pilus-related gene deletion strains. (M: DL5000 DNA marker; 1, 4, 7: negative control; 2, 5, 8: genome of WT A909; 3: ∆BP genome; 6: ∆AP1 genome; 9: ∆SrtC genome). **B** Electrophoresis diagram of PCR detection of the genetic stability of the pilus-related gene deletion strains. (M: DL5000 DNA marker; 1, 8, 15: negative control; 7, 14, 21: genome of WT A909; 2–6: genomes of different generations of ∆BP; 9–13: genomes of different generations of ∆AP1; 16–20: genomes of different generations of ∆SrtC). **C** Results of the determination of the bacterial growth curves of the strains WT A909, ∆BP, ∆AP1, and ∆SrtC. **D** Results of the detection of the bacterial morphology of the strains WT A909, ∆BP, ∆AP1, and ∆SrtC. Columns 1 and 2 show the scanning electron microscope observations; columns 3 and 4 show the transmission electron microscope observations. The roughness of the bacterial surface is indicated by the blue arrow “↑”, and the protrusion structure on the bacterial surface is indicated by the red arrow “↑”.

To investigate the effects of BP, AP1, and SrtC on the biological characteristics of A909, the gene deletion recombinant plasmids pSET4s-∆BP, pSET4s-∆AP1, and pSET4s-∆SrtC were electroporated into competent A909 cells. Suspected colonies that displayed loss of resistance were selected for PCR identification. The results revealed that after the deletion of the BP gene, the band size was 1293 bp (Figure 4A, Column 3); after the deletion of the AP1 gene, the band size was 1666 bp (Figure 4A, Column 6); and after the deletion of the SrtC gene, the band size was 1937 bp (Figure 4A, Column 9). These results were consistent with the expected fragment sizes. PCR identification and sequencing confirmed that the pilus-related gene deletion mutants ∆BP, ∆AP1, and ∆SrtC were successfully constructed.

Following continuous subculture, PCR identification was performed again, and the results matched the fragment sizes of the primary gene deletion mutants, indicating that the pilus-related genes could be stably inherited after deletion (Figure 4B). Bacterial growth curves were drawn and analysed, revealing no significant difference in growth rates between the WT A909 strain and the ∆BP, ∆AP1, and ∆SrtC strains. These findings suggest that the deletion of pilus-related genes did not affect the growth rate of A909 (Figure 4C).

The SEM and TEM results revealed that, compared with that of the WT A909 strain, the surface roughness of the ∆AP1 and ∆SrtC strains was altered. The ultrastructures of the bacteria were observed by TEM. For WT A909, protruding structures were present on the surface of the bacterial cells, and the outer layer structure of the cells was normal. In the ∆BP and ∆SrtC strains, protruding structures were also found on the surface of the bacterial cells, but the outer layer of the cells became thinner. For the ∆AP1 strain, no protrusions were observed on the surface of the bacterial cells, while the outer layer structure of the cells remained normal (Figure 4D).

**Figure 5:**
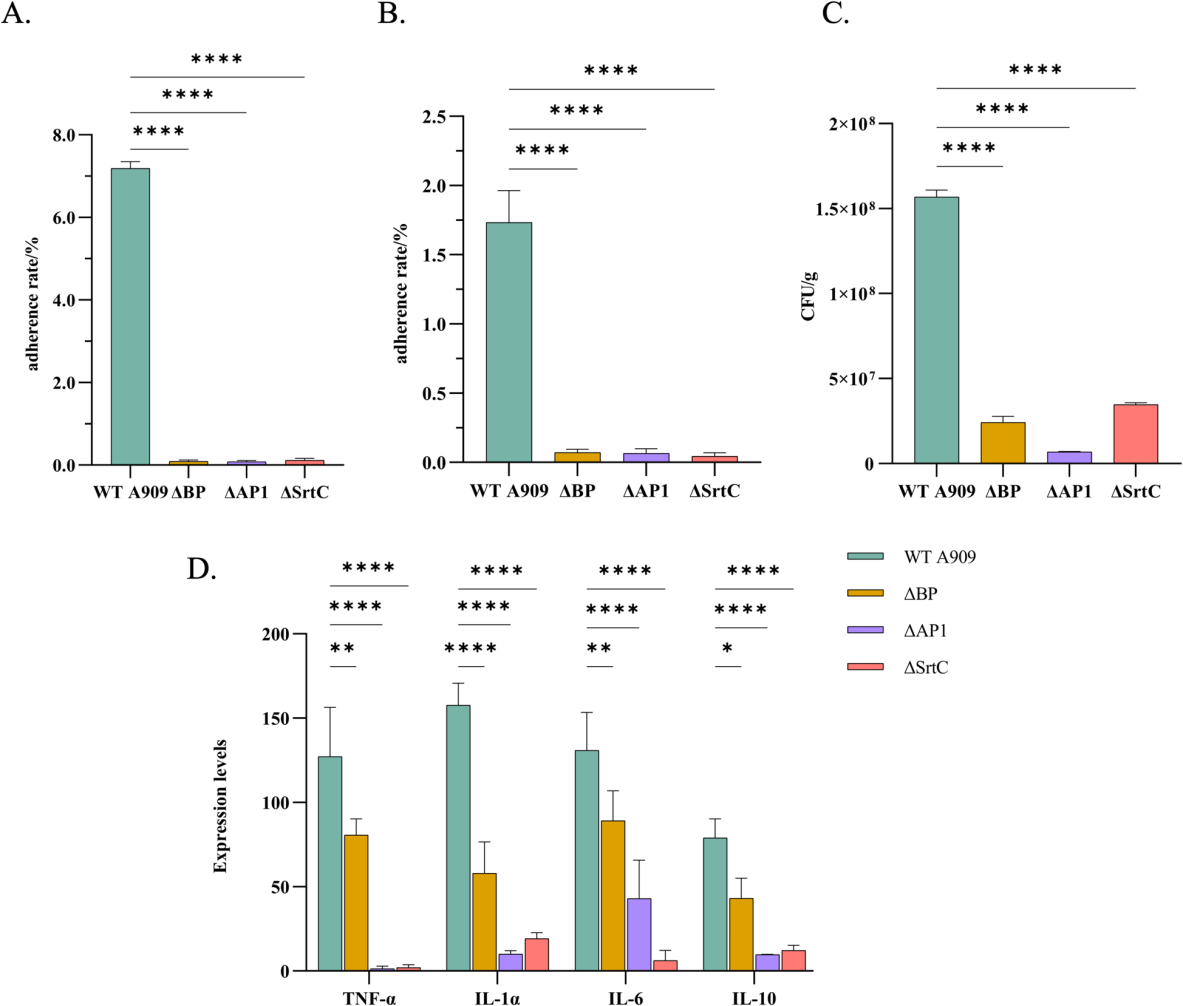
**Influence of pilus-related genes on the virulence of** ***S. agalactiae *A909.** **A** Determination of the ability of bacteria to adhere to EPH4-Ev cells. The data are presented as the mean ± standard deviation, ****P < 0.0001; one-way analysis of variance (ANOVA). **B** Determination of the ability of bacteria to adhere to MAC-T cells. The data are presented as mean ± standard deviation, **** P < 0.0001; one-way ANOVA. **C** Determination of the bacterial load in mouse mammary gland tissue. CFU: Colony forming unit. The data are presented as the mean ± standard deviation, ** P < 0.01; one-way ANOVA. **D** Detection of the expression levels of inflammatory factors in mammary gland tissue by qPCR. *IL* Interleukin, *TNF* Tumor necrosis factor. The data are presented as mean ± standard deviation, * P < 0.05; ** P < 0.01; **** P < 0.0001; one-way ANOVA.

To explore the effects of the pilus-related genes BP, AP1, and SrtC on the virulence of A909, the adhesion rates of the ∆BP, ∆AP1, ∆SrtC strains and WT A909 to EPH4-Ev and MAC-T cells were determined. Moreover, the bacterial load in the mammary tissue of the mice and the expression levels of inflammatory factors were also detected. The results of the adhesion rate determination revealed that in EPH4-Ev cells, the adhesion abilities of the ∆BP (0.09 ± 0.03)%, ∆AP1 (0.08 ± 0.03)%, and ∆SrtC (0.12 ± 0.04) groups were significantly lower than those of the WT A909 group (7.19 ± 0.16) (Figure 5A). In MAC-T cells, the adhesion abilities of the ∆BP (0.072 ± 0.02)%, ∆AP1 (0.065 ± 0.03%), and ∆SrtC (0.044 ± 0.02)% groups were also lower than those of the WT A909 group (1.73 ± 0.23)% (Figure 5B). The detection of the bacterial load in the mammary tissue of the mice revealed that the bacterial loads in the mammary tissue of the mice in the ∆BP (2.43 × 10^7^ ± 2.47 × 10^6^) CFU/g, ∆AP1 (6.95 × 10^6 ^± 4 × 10^4^) CFU/g, and ∆SrtC (3.47 × 10^7^ ± 8 × 10^5^) CFU/g groups were significantly lower than those in the WT A909 group (1.57 × 10^8 ^± 4.01 × 10^6^) CFU/g (Figure 5C). The results of the quantitative analysis of inflammatory factors revealed that the expression levels of IL-1α, IL-6, IL-10, and TNF-α in the mammary tissue of the mice in the ∆BP, ∆AP1, and ∆SrtC groups were lower than those in the WT A909 group (Figure 5D).

On the basis of the above experimental data, the deletion of the pilus-related genes BP, AP1, and SrtC significantly reduced the adhesion ability of A909 to cells, decreased the bacterial load in the mammary tissue of mice, and reduced the expression levels of inflammatory factors. These findings indicate that these pilus-related genes play important roles in the virulence of A909 and that their deletion can lead to a decrease in the virulence of A909.

### Effects of pilus-related genes on host cell gene expression differences

Differentially expressed genes (DEGs) were screened according to the general standard threshold (fold change = twofold, Padj ≤ 0.05). Among them, genes with Log FC (fold change) ≥ 1 and Padj ≤ 0.05 were defined as upregulated genes, whereas genes with Log FC ≤ -1 and Padj ≤ 0.05 were defined as downregulated genes. Genes with Log FC values between − 1 and 1 were considered genes with no change in expression level.

**Figure 6:**
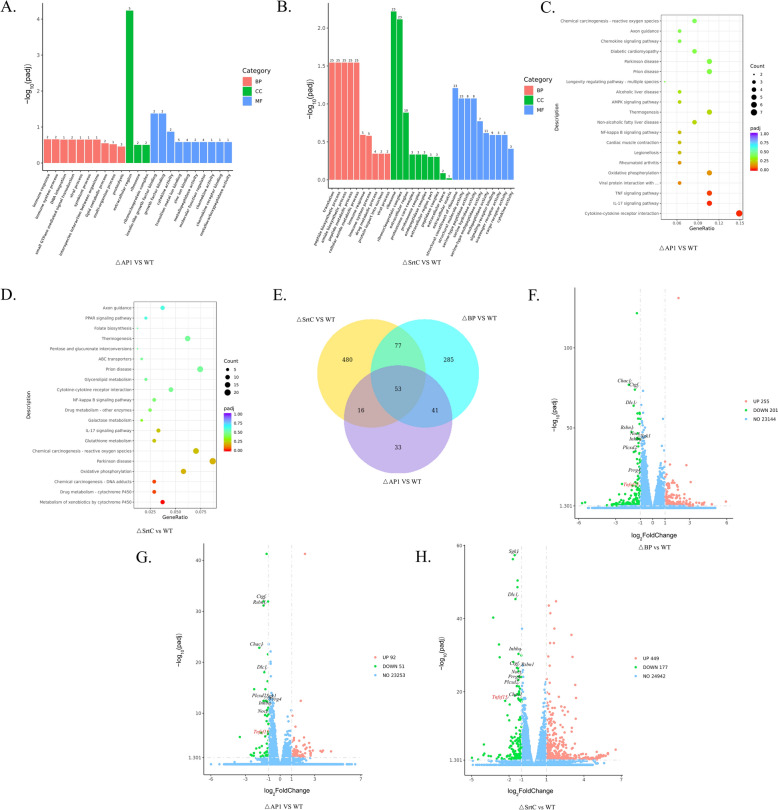
**Influence of pilus-related genes on the differential gene expression of host cells.** **A**, **B** Histogram of the results of the GO functional enrichment analysis of DEGs in host cells after EPH4-Ev cells were infected with ∆AP1 and ∆SrtC, compared with those in host cells infected with WT A909. Red represents biological processes, green represents cellular components, and blue represents molecular functions. The horizontal axis (X-axis) represents different biological function annotation categories; the vertical axis (Y-axis) represents the *P* value after multiple hypothesis testing correction. After the negative logarithm is taken, a larger value indicates more significant differential expression of the gene. **C**, **D** Bubble plot of KEGG metabolic pathway enrichment analysis of DEGs in host cells after EPH4-Ev cells were infected with ∆AP1 and ∆SrtC, compared with those in host cells infected with WT A909. The color represents the corrected *P* value. From purple to red, the more significant the enrichment result is; the size of the bubble represents the number of enriched genes. **E** Venn diagram of the DEGs commonly expressed among the three comparison groups (∆BP vs WT A909, ∆AP1 vs WT A909, and ∆SrtC vs WT A909). **F**–**H** Volcano plots of DEGs in host cells after EPH4-Ev cells were infected with ∆BP, ∆AP1, and ∆SrtC, compared with those in host cells infected with WT A909. The horizontal axis (X-axis) represents the log_2_-fold change in gene expression; the vertical axis (Y-axis) represents the *P* value after multiple hypothesis testing correction. After the negative logarithm is taken, a larger value indicates more significant differential expression of the gene. The red dots represent upregulated DEGs, the green dots represent downregulated DEGs, and the blue dots represent non-DEGs.

To further explore the characteristics of the DEGs in terms of molecular functions and metabolic pathways, this study employed Gene Ontology (GO) functional enrichment analysis and Kyoto Encyclopedia of Genes and Genomes (KEGG) metabolic pathway enrichment analysis. The results of the GO functional enrichment analysis revealed that there was no significant enrichment of DEGs in the ∆BP vs WT A909 group. Compared with the WT group, the ∆AP1 group was significantly enriched in the extracellular region, insulin-like growth factor-binding protein, and growth factor binding (Figure 6A), which implies that it may play important roles in intercellular communication, growth regulation, and other aspects. Compared with the WT A909 group, the ∆SrtC group was significantly enriched in processes related to translation, peptide biosynthesis, and metabolism (Figure 6B), indicating that it has a crucial impact on basic substance synthesis and metabolism in bacteria. KEGG metabolic pathway enrichment analysis revealed that there was no significant enrichment of DEGs in the ∆BP group compared with the WT A909 group. Compared with the WT group, the ∆AP1 group was significantly enriched in immune-related pathways, such as cytokine–cytokine–receptor interactions (Figure 6C), which are closely related to the immune response and reflect its potential role in host immune regulation. Compared with the WT A909 group, the ∆SrtC group was significantly enriched in pathways related to drug metabolism and chemical carcinogenesis (Figure 6D).

Through volcano plot analysis, the DEGs in these three groups were clearly presented. Among them, 53 DEGs were common to all three groups (Figure 6E). Functional analysis revealed that these genes are extensively involved in metabolic regulatory pathways, cellular physiological activity regulation, and signal transduction networks and play crucial roles in maintaining cellular homeostasis and physiological functions. We selected the 10 most significantly differentially expressed genes from the 53 DEGs for verification of the accuracy of the transcriptome results (Figures 6F–H). qPCR analysis revealed that the results were consistent with the trends observed in the transcriptome data, indicating that the transcriptome results are reliable (Additional file 2). Among these differential genes, *Tnfsf15* attracted our attention. *Tnfsf15* is a member of the TNF superfamily and plays a primary role in immune responses. It is involved in the regulation of cell apoptosis, inflammatory responses, and the overall function of the immune system, playing a pivotal role in the activation of immune cells and the modulation of inflammation. In light of the inflammatory phenotype of mastitis induced by A909, we hypothesize that this gene is closely associated with the pilus-related gene cluster of A909 in the pathogenesis of mastitis. Preliminary validation of this hypothesis has been carried out.

### Effect of Tnfsf15 gene expression levels on the adhesion of S. agalactiae A909 to EPH4-Ev cells

**Figure 7:**
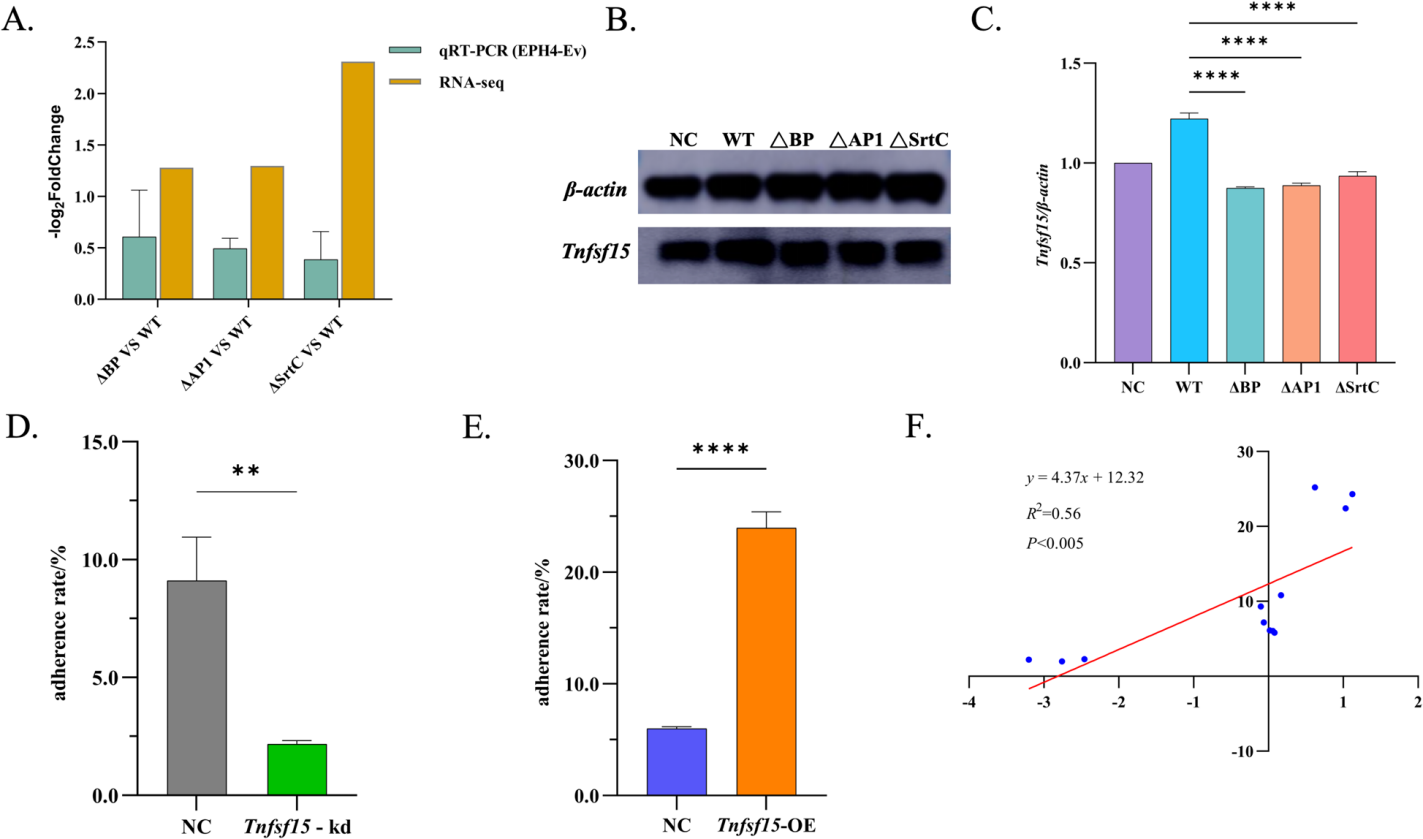
**Effects of Tnfsf15 gene regulation on the adhesion of** ***S. agalactiae* A909 to EPH4-Ev cells.A** Histogram for verifying the expression level of *Tnfsf15* in host cells. Log_2_FC indicates the log_2_-fold change. A log_2_FC > 0 indicates that the expression of the *Tnfsf15* gene in the ∆BP, ∆AP1, and ∆SrtC treatment groups was upregulated compared with that in the WT A909 control group; conversely, it was downregulated. The larger the absolute value of Log_2_FC is, the more significant the difference in gene expression between the two samples. **B** Protein expression of *Tnfsf15* in EPH4-Ev cells infected with WT, BP, AP1 or SrtC. **C** Band gray value ratio of the western blot. All the results were normalized to those of β-actin; **** P < 0.0001. **D**, **E** Adhesion rates of A909 and EPH4-Ev cells under different *Tnfsf15* transfection conditions. The data are presented as the mean ± standard deviation, **** P < 0.0001; Student’s *t* test. **F** Scatter plot and linear regression fitting of the correlation between the *Tnfsf15* mRNA expression level and the bacterial adhesion rate. Scatter points represent the corresponding *Tnfsf15* mRNA expression levels and bacterial adhesion rates in different samples. Red straight line: linear regression fitting line used to show the linear relationship trend between the *Tnfsf15* mRNA expression level and the bacterial adhesion rate.

To further investigate the role of the *Tnfsf15* gene, a host gene, in the pathogenic process of the pilus-related gene cluster, we conducted a preliminary exploration. First, we assessed the transcriptional and protein expression levels of *Tnfsf15* in EPH4-Ev cells treated with the WT, ΔBP, ΔAP1, and ΔSrtC strains by quantitative PCR and western blotting. Compared with those in the WT A909 group, the transcription and protein expression of the *Tnfsf15* gene in the ∆BP, ∆AP1, and ∆SrtC groups all tended to decrease, which was consistent with the transcriptomic data (Figures 7A–C). Next, we altered the transcription levels of *Tnfsf15* via RNA interference and overexpression plasmids and examined the relationships between *Tnfsf15* expression and the adhesion of A909 and EPH4-Ev cells. The results revealed that the adhesion rate of A909 cells in the *Tnfsf15*-kd group was 2.17 ± 0.15%, which was significantly lower than that in the NC group (9.11 ± 1.84%, *P* < 0.001) (Fig. 7D). In contrast, the adhesion rate of A909 cells in the *Tnfsf15*-OE group was 23.97 ± 1.43%, which was significantly greater than that in the NC group (6 ± 0.16%, *P* < 0.0001) (Figure 7E). The verification results of *Tnfsf15* gene knockdown and overexpression are shown in Additional file 3. Correlation analysis revealed a significant positive correlation between *Tnfsf15* expression levels and the number of A909 adherent cells (r = 0.75, *P* < 0.005) (Figure 7F). These findings suggest that the pilus-related gene cluster may regulate the adhesion and invasion ability of A909 cells by modulating the expression of *Tnfsf15*, thereby influencing disease progression. However, the exact regulatory mechanisms remain to be further investigated.

## Discussion

*S. agalactiae* adheres to extracellular matrix proteins on the surface of mammary epithelial cells via adhesion-related virulence genes, which represents the primary step in the development of bovine mastitis caused by this bacterium. In this study, by leveraging the whole-genome transposon mutant library of A909 and integrating it with the EPH4-Ev cell adhesion model, a pilus gene cluster that is significantly associated with its virulence was identified. Moreover, the roles of the BP, AP1, and SrtC genes within the gene cluster in bacterial virulence were verified*.*

In this study, A909 demonstrated a high adhesion rate to both EPH4-Ev cells and MAC-T cells. Since cell adhesion is a crucial initial step in the bacterial infection of host cells, this finding suggests that A909 has the ability to invade mammary epithelial cells. Although the adhesion rates between the two cell types differed, both findings collectively confirmed the cell adhesion ability of A909, which was evident in cells derived from different sources. In the established mouse mastitis model, the ability of A909 to adhere to EPH4-Ev cells translated into a tangible infection effect, successfully triggering typical inflammatory responses. Considering the similarities in cell composition and physiological functions between bovine and murine mammary glands, along with the ability of A909 to adhere to both MAC-T and EPH4-Ev cells and the induction of inflammatory responses in mice, it is reasonable to hypothesize that in cattle, A909 may initiate the infection process through a similar adhesion mechanism. Although notable species differences exist between mice and cattle, from the perspective of the basic mechanisms of cell adhesion and inflammatory responses, the mouse mastitis model can partially simulate the pathogenesis of bovine mastitis [25–27]. The mouse model offers significant advantages, such as low cost, ease of use, and a short experimental timeline, providing valuable data for studying the infection mechanism of A909 within limited resources and time. Therefore, the mouse mastitis model, as an alternative for bovine mastitis research, is scientifically feasible.

A gene cluster region closely related to the adhesion and invasion of A909 was identified by infecting EPH4-Ev cells with a mutant library. This region contains key proteins essential for pilus assembly and expression, including backbone proteins, accessory proteins, and sortases. Compared with previous studies on pilus-related genes of *S. agalactiae* and other Gram-positive bacteria [28–30], this study is the first to identify an insertion mutation at the stop codon of SAK_RS11705, which fused the originally independent SAK_RS11705, SAK_RS11690 and SAK_RS11695 into one gene. This discovery highlights the dynamic changes in gene structure. Additionally, the high homology between the proteins encoded by the related genes and proteins with known functions provides further enrichment of the functional information on the pilus gene family of *S. agalactiae* and other Gram-positive bacteria.

On the basis of the above findings, this study further constructed deletion mutants of pilus-related genes (BP, AP1, and SrtC) to systematically investigate the roles of these genes in bacterial physiological functions and the pathogenic process. By integrating existing research results, we have gained a more in-depth understanding of the functions of pilus-related genes and their regulatory mechanisms in host‒pathogen interactions.

From the perspective of gene function conservation, the deletion of the AP1 gene led to the disappearance of the protrusion structures on the bacterial surface, which is highly consistent with the classical understanding that accessory proteins in Gram-positive bacteria are involved in the assembly of the pilus tip [17]. The changes in the outer layer structure of the cells caused by the deletion of the BP and SrtC genes are consistent with the functional characteristics of BP as a backbone protein maintaining the spatial stability of the pili and SrtC as a sortase mediating the covalent cross-linking of protein subunits. This phenomenon has also been confirmed in the study of the impact of the deletion of SrtA in *Staphylococcus aureus* on the assembly of cell wall-anchored proteins [31], further indicating that pilus-related genes maintain the structural integrity of pili through synergistic effects, thereby influencing the basic physiological functions of bacteria.

In terms of the pathogenic mechanism, the deletion of pilus-related genes (BP, AP1, and SrtC) resulted in a significant decrease in the virulence of A909, and this result can be explained from multiple dimensions. As the core component for bacterial adhesion to host cells, the structural defects of the pili directly weaken the specific binding ability of the bacteria to receptors such as fibronectin and laminin on the surface of host cells, blocking the initiation of infection, which is consistent with previous research conclusions on the adhesion mechanism of *S. agalactiae* pili [16]. An abnormal pilus structure may alter the exposure pattern of bacterial surface antigenic epitopes, making bacteria more easily recognized and eliminated by the host immune system. This phenomenon is similar to the situation in which the pilus tip adhesin PilA during the infection process of GBS not only facilitates bacterial adhesion but also enables immune evasion by manipulating the host immune response. The crucial role of pili in biofilm formation should not be overlooked. Research results showing that the absence of pili in pathogenic bacteria such as *Enterococcus faecalis* leads to biofilm formation disorders provide theoretical support for the decreased colonization ability of the gene deletion mutants in the mouse infection model of this experiment [32–34].

The *Tnfsf15* gene may play a critical role in the inflammatory damage caused by nonlactating *S. agalactiae* in mastitis. Studies have shown that *Tnfsf15* is a cytokine secreted by immune cells that regulates the activity of immune cells and induces localized immune damage during inflammatory responses. In this study, through transcriptomic analysis, we found that the expression of *Tnfsf15* is closely associated with the pathogenicity of the pilus-associated gene cluster of nonlactating *S. agalactiae*. Pilus-associated gene clusters are typically involved in bacterial adhesion and invasion, whereas *Tnfsf15*, as a key regulator of the inflammatory response, may exert its effect by inducing the release of inflammatory cytokines [35], thereby enhancing the immune system’s response to bacterial infection and ultimately leading to inflammation and functional loss in mammary tissue. Furthermore, *Tnfsf15* has been shown to play an important proinflammatory role in various bacterial infections. Studies indicate that *Tnfsf15* activates the NF-κB pathway through receptor interactions, thereby enhancing the local immune response [36]. During mastitis caused by nonlactating *S. agalactiae*, *Tnfsf15* may promote localized inflammation by modulating this immune pathway, leading to mammary cell damage and tissue necrosis. Notably, *Tnfsf15* is closely related to the immune microenvironment of the mammary gland, and its upregulation in various inflammatory models is often accompanied by severe tissue damage. However, this is only our preliminary hypothesis, and the molecular connection between *Tnfsf15* and the pilus-associated gene cluster of nonlactating *S. agalactiae* in mastitis will be further elucidated in subsequent studies.

In conclusion, this study successfully identified a novel gene cluster responsible for pilus synthesis in the A909 strain of *S. agalactiae* and established a clear correlation between pilus-associated genes (BP, AP1, and SrtC) and bacterial virulence. By integrating transcriptome sequencing with functional analyses involving *Tnfsf15* gene knockdown and overexpression, we further revealed that *Tnfsf15* plays a pivotal role in mediating host‒pathogen interactions through specific signalling pathways. These findings not only bridge a critical knowledge gap regarding the genetic basis of pilus synthesis in *S. agalactiae* but also reveal a new regulatory axis contributing to bacterial pathogenicity. Overall, this work provides a foundation for future mechanistic studies and opens new avenues for the development of targeted interventions against *S. agalactiae* infections.

## Supplementary Information

**Additional file 1. Primer sequences for quantitative real-time polymerase chain reaction.**

**Additional file 2. Comparison of the Fold Changes in the Expression of DEGs between Gene Deletion Strains (**∆**BP, **∆**AP1, **∆**SrtC) and the WT as Detected by Transcriptome Sequencing and qRT-PCR**. RNA-seq: Results of transcriptome sequencing.

**Additional file 3. Verification of Tnfsf15 gene knockdown and overexpression. (**A) Effect of RNA interference on the mRNA expression of the *Tnfsf15* gene. NC: small interfering RNA negative control; *Tnfsf*15-kd: treatment group with small interfering RNA targeting the *Tnfsf15* gene. *** P < 0.001; Student’s *t* test. (B) Effects of overexpression on the mRNA expression of the *Tnfsf15* gene. NC: negative control group; *Tnfsf15*-OE: treatment group with the *Tnfsf15* gene overexpression vector. The log_2_FC represents the log_2_-fold change. **P < 0.01; Student’s *t* test. (C) Verification of the effects of the RNA interference and overexpression plasmids on *Tnfsf15* protein expression. (D) Band gray value ratios of the western blot data, with all the results normalized to those of β-actin.

## Data Availability

The data of the results in this study are available from the authors upon reasonable request.

## References

[1] Delannoy CM, Crumlish M, Fontaine MC, Pollock J, Foster G, Dagleish MP, Turnbull JF, Zadoks RN (2013) Human *Streptococcus agalactiae* strains in aquatic mammals and fish. BMC Microbiol 13:41 23419028 10.1186/1471-2180-13-41 PMC3585737

[2] Raabe VN, Shane AL (2019) Group B streptococcus (*Streptococcus agalactiae*). Microbiol Spectr 7:10.1128/microbiolspec.gpp3-0007-2018 30900541 10.1128/microbiolspec.gpp3-0007-2018 PMC6432937

[3] Asfaw M, Negash A (2017) Review on impact of bovine mastitis in dairy production. Adv Biol Res 11:126–131

[4] Krishnamoorthy P, Suresh KP, Jayamma KS, Shome BR, Patil SS, Amachawadi RG (2021) An understanding of the global status of major bacterial pathogens of milk concerning bovine mastitis: a systematic review and meta-analysis (Scientometrics). Pathogens 10:545 33946571 10.3390/pathogens10050545 PMC8147236

[5] Kabelitz T, Aubry E, van Vorst K, Amon T, Fulde M (2021) The role of *Streptococcus* spp. in bovine mastitis. Microorganisms 9:1497 34361932 10.3390/microorganisms9071497 PMC8305581

[6] Cheng WN, Han SG (2020) Bovine mastitis: risk factors, therapeutic strategies, and alternative treatments—a review. Asian-Australas J Anim Sci 33:1699–1713 32777908 10.5713/ajas.20.0156 PMC7649072

[7] Paramasivam R, Gopal DR, Dhandapani R, Subbarayalu R, Elangovan MP, Prabhu B, Veerappan V, Nandheeswaran A, Paramasivam S, Muthupandian S (2023) Is AMR in dairy products a threat to human health? An updated review on the origin, prevention, treatment, and economic impacts of subclinical mastitis. Infect Drug Resist 16:155–178 36636377 10.2147/IDR.S384776 PMC9831082

[8] Maity S, Ambatipudi K (2021) Mammary microbial dysbiosis leads to the zoonosis of bovine mastitis: a One-Health perspective. FEMS Microbiol Ecol 97:fiaa241 10.1093/femsec/fiaa241 33242081

[9] Torres G, Macias D, Reyes-Vélez J, Rios-Agudelo P, Caraballo-Guzmán A (2023) *Streptococcus agalactiae* virulence factors isolated from bovine mastitis and antibiotic treatment response. J Appl Microbiol 134:lxad116 37353927 10.1093/jambio/lxad116

[10] Zastempowska E, Twarużek M, Grajewski J, Lassa H (2022) Virulence factor genes and cytotoxicity of *Streptococcus agalactiae* isolated from bovine mastitis in Poland. Microbiol Spectr 10:e0222421 35608349 10.1128/spectrum.02224-21 PMC9241884

[11] Liu X, Pang X, Wu Y, Wu Y, Xu L, Chen Q, Niu J, Zhang X (2023) New insights into the lactic acid resistance determinants of *Listeria monocytogenes* based on transposon sequencing and transcriptome sequencing analyses. Microbiol Spectr 11:e0275022 36541787 10.1128/spectrum.02750-22 PMC9927151

[12] Kawakami K, Largaespada DA, Ivics Z (2017) Transposons as tools for functional genomics in vertebrate models. Trends Genet 33:784–801 28888423 10.1016/j.tig.2017.07.006 PMC5682939

[13] Tsai JC, Loh JM, Clow F, Lorenz N, Proft T (2017) The Group A streptococcus serotype M2 pilus plays a role in host cell adhesion and immune evasion. Mol Microbiol 103:282–298 27741558 10.1111/mmi.13556

[14] Cozzi R, Malito E, Lazzarin M, Nuccitelli A, Castagnetti A, Bottomley MJ, Margarit I, Maione D, Rinaudo CD (2015) Structure and assembly of group B streptococcus pilus 2b backbone protein. PLoS One 10:e0125875 25942637 10.1371/journal.pone.0125875 PMC4420484

[15] Maisey HC, Quach D, Hensler ME, Liu GY, Gallo RL, Nizet V, Doran KS (2008) A group B streptococcal pilus protein promotes phagocyte resistance and systemic virulence. FASEB J 22:1715–1724 18198218 10.1096/fj.07-093963 PMC2721339

[16] Périchon B, Guignot J, Szili N, Gao C, Poyart C, Trieu-Cuot P, Dramsi S (2019) Insights into *Streptococcus agalactiae* PI-2b pilus biosynthesis and role in adherence to host cells. Microbes Infect 21:99–103 30419351 10.1016/j.micinf.2018.10.004

[17] Telford JL, Barocchi MA, Margarit I, Rappuoli R, Grandi G (2006) Pili in gram-positive pathogens. Nat Rev Microbiol 4:509–519 16778837 10.1038/nrmicro1443

[18] Khare B, Krishnan V, Rajashankar K, I-Hsiu H, Xin M, Ton-That H, Narayana S (2011) Structural differences between the *Streptococcus agalactiae* housekeeping and pilus-specific sortases: SrtA and SrtC1. PLoS One 6:e22995 21912586 10.1371/journal.pone.0022995 PMC3166054

[19] Chang C, Ramirez NA, Bhat AH, Nguyen MT, Kumari P, Ton-That H, Das A, Ton-That H (2024) Biogenesis and functionality of sortase-assembled pili in gram-positive bacteria. Annu Rev Microbiol 78:403–423 39141696 10.1146/annurev-micro-112123-100908

[20] Kadiyska T, Tourtourikov I, Popmihaylova AM, Kadian H, Chavoushian A (2018) Role of TNFSF15 in the intestinal inflammatory response. World J Gastrointest Pathophysiol 9:73 30809418 10.4291/wjgp.v9.i4.73 PMC6384511

[21] Valatas V, Kolios G, Bamias G (2019) TL1A (TNFSF15) and DR3 (TNFRSF25): a co-stimulatory system of cytokines with diverse functions in gut mucosal immunity. Front Immunol 10:421466 10.3389/fimmu.2019.00583 PMC6445966 30972074

[22] Richard AC, Peters JE, Savinykh N, Lee JC, Hawley ET, Meylan F, Siegel RM, Lyons PA, Smith KG (2018) Reduced monocyte and macrophage *TNFSF15/TL1A* expression is associated with susceptibility to inflammatory bowel disease. PLoS Genet 14:e1007458 30199539 10.1371/journal.pgen.1007458 PMC6130856

[23] Kokkotis G, Bamias G (2022) *TL1A* as a therapeutic target in inflammatory bowel disease. Expert Rev Clin Immunol 18:551–555 35507314 10.1080/1744666X.2022.2074401

[24] Liu R, Zhang P, Su Y, Lin H, Zhang H, Yu L, Ma Z, Fan H (2016) A novel suicide shuttle plasmid for *Streptococcus**suis* serotype 2 and Streptococcus equi ssp. zooepidemicus gene mutation. Sci Rep 6:27133 27256117 10.1038/srep27133 PMC4891806

[25] Brouillette E, Malouin F (2005) The pathogenesis and control of *Staphylococcus aureus*-induced mastitis: study models in the mouse. Microbes Infect 7:560–568 15777742 10.1016/j.micinf.2004.11.008

[26] Notebaert S, Meyer E (2006) Mouse models to study the pathogenesis and control of bovine mastitis. A review. Vet Q 28:2–13 16605156 10.1080/01652176.2006.9695201

[27] Ingman WV, Glynn DJ, Hutchinson MR (2015) Mouse models of mastitis–how physiological are they? Int Breastfeed J 10:1–6 25848399 10.1186/s13006-015-0038-5 PMC4386103

[28] Proft T, Baker E (2009) Pili in Gram-negative and Gram-positive bacteria—structure, assembly and their role in disease. Cell Mol Life Sci 66:613–635 18953686 10.1007/s00018-008-8477-4 PMC11131518

[29] Krishnan V, Dwivedi P, Kim BJ, Samal A, Macon K, Ma X, Mishra A, Doran KS, Ton-That H, Narayana SV (2013) Structure of *Streptococcus agalactiae* tip pilin GBS104: a model for GBS pili assembly and host interactions. Acta Crystallogr D Biol Crystallogr 69:1073–1089 23695252 10.1107/S0907444913004642 PMC3663123

[30] Rosini R, Rinaudo CD, Soriani M, Lauer P, Mora M, Maione D, Taddei A, Santi I, Ghezzo C, Brettoni C (2006) Identification of novel genomic islands coding for antigenic pilus-like structures in *Streptococcus agalactiae*. Mol Microbiol 61:126–141 16824100 10.1111/j.1365-2958.2006.05225.x

[31] Lee J, Choi JH, Lee J, Cho E, Lee YJ, Lee HS, Oh KB (2024) Halenaquinol blocks staphylococcal protein A anchoring on cell wall surface by inhibiting sortase A in *Staphylococcus aureus*. Mar Drugs 22:266 38921577 10.3390/md22060266 PMC11204543

[32] Sillanpää J, Nallapareddy SR, Singh KV, Prakash VP, Fothergill T, Ton-That H, Murray BE (2010) Characterization of the *ebp*_*fm*_ pilus-encoding operon of *Enterococcus faecium* and its role in biofilm formation and virulence in a murine model of urinary tract infection. Virulence 1:236–246 20676385 10.4161/viru.1.4.11966 PMC2910428

[33] Singh KV, Nallapareddy SR, Murray BE (2007) Importance of the *ebp* (endocarditisand biofilm-associated pilus) locus in the pathogenesis of *Enterococcus faecalis* ascending urinary tract infection. J Infect Dis 195:1671–1677 17471437 10.1086/517524 PMC2680192

[34] Montealegre MC, La Rosa SL, Roh JH, Harvey BR, Murray BE (2015) The *Enterococcus faecalis EbpA* pilus protein: attenuation of expression, biofilm formation, and adherence to fibrinogen start with the rare initiation codon ATT. mBio 6:e00467-15 26015496 10.1128/mBio.00467-15 PMC4447247

[35] Jin S, Chin J, Seeber S, Niewoehner J, Weiser B, Beaucamp N, Woods J, Murphy C, Fanning A, Shanahan F (2013) *TL1A/TNFSF15* directly induces proinflammatory cytokines, including TNFα, from CD3+CD161+ T cells to exacerbate gut inflammation. Mucosal Immunol 6:886–899 23250276 10.1038/mi.2012.124

[36] Xu WD, Li R, Huang AF (2022) Role of *TL1A* in inflammatory autoimmune diseases: a comprehensive review. Front Immunol 13:891328 35911746 10.3389/fimmu.2022.891328 PMC9329929

