# Supplementary material for: A novel pilus-associated gene cluster is implicated in Streptococcus agalactiae virulence
Source: Vet Res. 2025 Jul 3;56:135. doi: 10.1186/s13567-025-01567-z (PMC12224707; doi:10.1186/s13567-025-01567-z)
Supplement: Supplementary file 1 — Additional file 1. Primer sequences for quantitative real-time polymerase chain reaction. [file 13567_2025_1567_MOESM1_ESM.docx]

**Additional file 1. Primer sequence of quantitative real-time polymerase chain reaction**

| Gene name | Primer sequence |
| --- | --- |
| Chac1- qF | CTTGAAGACCGTGAGGGCTG |
| Chac1- qR | GGTACTTCAGGGCCTCGTTC |
| Noct- qF | CGTCCCCGAACAGTGAGTT |
| Noct- qR | GGATCGATGGGCTCCAGATG |
| Prrg4- qF | CCGGGGACACAGTTTGTTTG |
| Prrg4- qR | CAATGAGGAACTGCGAGGGT |
| Ctgf- qF | GGCCTCTTCTGCGATTTCG |
| Ctgf- qR | GCAGCTTGACCCTTCTCGG |
| Plcxd2- qF | ACTTTGACCTACGGGTGTCTT |
| Plcxd2- qR | CTCCATCAACCCATCCCAAAC |
| Dlc1-qF | GGACACCATGATCCTAACACAAA |
| Dlc1-qR | AGCGCAATATCAACAGGGAAC |
| Sgk1-qF | TCAGAGCGGAATGTTCTGTTG |
| Sgk1-qR | AGCGGTCTGGAATGAGAAGTG |
| Tnfsf15-qF | GAGAGCACACCTGACAATTAAGA |
| Tnfsf15-qR | TGTGGTCCCTCGGAATGTGAT |
| Inhba-qF | TCCGAAGGATGGACCTAACTC |
| Inhba-qR | GCTTTCTGATCGCGTTGAGAAG |
| Rsbn1-qF | CCAAGCATAAGGGCCACAAG |
| Rsbn1-qR | CTCCTTTCGCCGTTTTCCTG |
| gapdh-qF | GCGACTTCAACAGCAACTCCC |
| gapdh-qR | CACCCTGTTGCTGTAGCCGTA |
