# Supplementary material for: A novel pilus-associated gene cluster is implicated in Streptococcus agalactiae virulence
Source: Vet Res. 2025 Jul 3;56:135. doi: 10.1186/s13567-025-01567-z (PMC12224707; doi:10.1186/s13567-025-01567-z)
Supplement: Supplementary file 2 — Additional file 2. Comparison of the Fold Changes in the Expression of DEGs between Gene Deletion Strains (∆BP, ∆AP1, ∆SrtC) and the WT as Detected by Transcriptome Sequencing and qRT-PCR. RNA-seq: Results of transcriptome sequencing. [file 13567_2025_1567_MOESM2_ESM.docx]

**Additional file 2. Comparison of the Fold Changes in the Expression of DEGs between Gene Deletion Strains (△BP, △AP1, △SrtC) and the WT as Detected by Transcriptome Sequencing and qRT-PCR.** RNA-seq: Results of transcriptome sequencing.

| gene | △BP vs WT | | △AP1 vs WT | | △SrtC vs WT | |
| --- | --- | --- | --- | --- | --- | --- |
|  | RNA-seq | qRT-PCR | RNA-seq | qRT-PCR | RNA-seq | qRT-PCR |
| Chac1 | -1.94 | -0.84 | -1.81 | -0.67 | -1.22 | -1.43 |
| Noct | -1.27 | -0.75 | -1.31 | -0.80 | -1.24 | -1.25 |
| Prrg4 | -1.20 | -0.10 | -1.05 | -0.07 | -1.26 | -0.61 |
| Ctgf | -1.45 | -1.30 | -1.45 | -1.28 | -1.37 | -2.07 |
| Plcxd2 | -1.54 | -0.12 | -1.45 | -0.33 | -1.38 | -0.69 |
| Dlc1 | -1.55 | -0.93 | -1.37 | -0.34 | -1.49 | -0.76 |
| Sgk1 | -1.15 | -0.95 | -1.25 | -0.62 | -1.55 | -1.29 |
| Inhba | -1.10 | -0.39 | -1.03 | -0.70 | -1.24 | -0.80 |
| Rsbn1 | -1.71 | -0.19 | -1.44 | -0.55 | -1.28 | -0.64 |
| Tnfsf15 | -1.28 | -0.61 | -1.30 | -0.49 | -2.31 | -0.39 |
