# Supplementary figures and images for: A novel pilus-associated gene cluster is implicated in Streptococcus agalactiae virulence
Source: Vet Res. 2025 Jul 3;56:135. doi: 10.1186/s13567-025-01567-z (PMC12224707; doi:10.1186/s13567-025-01567-z)

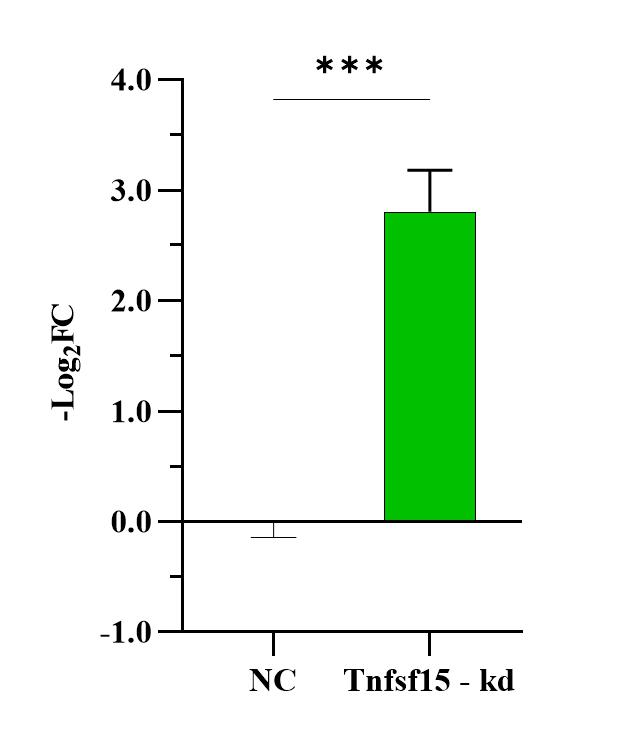

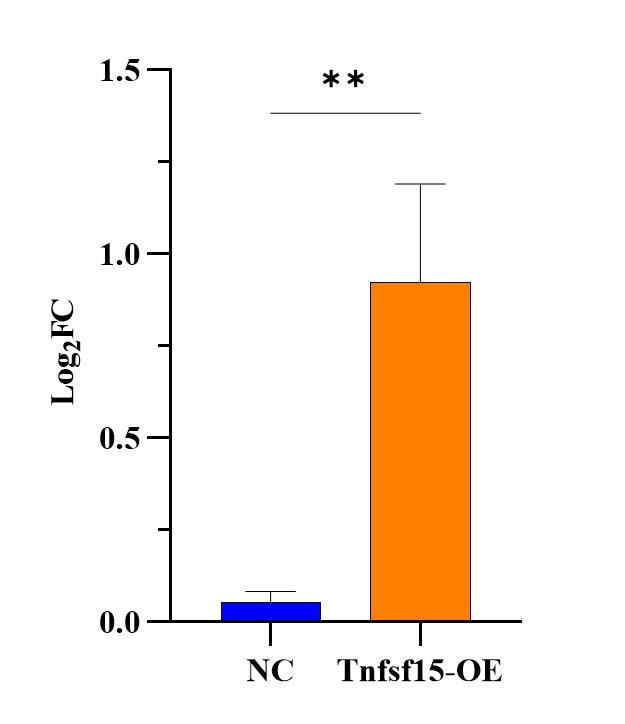

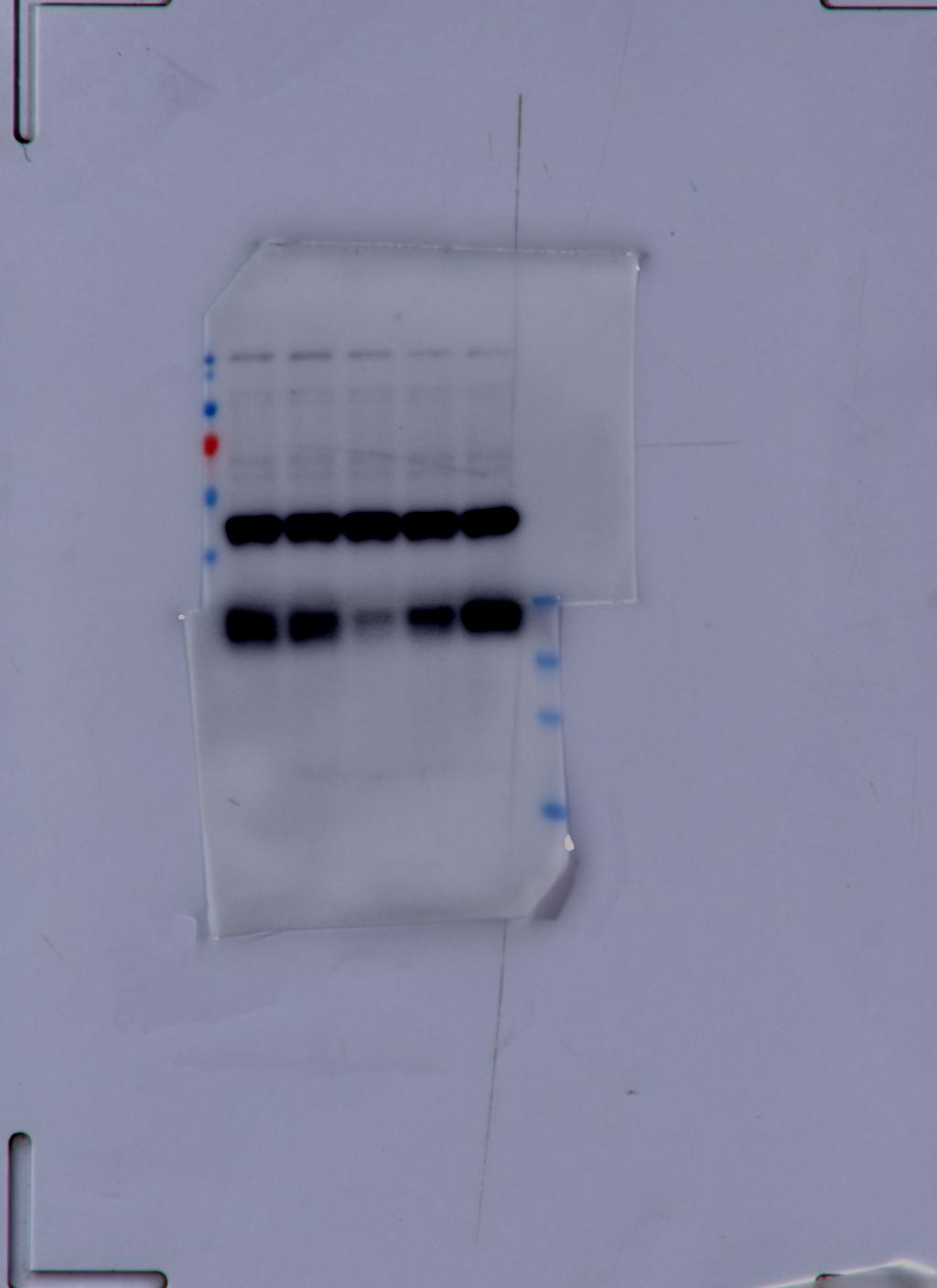

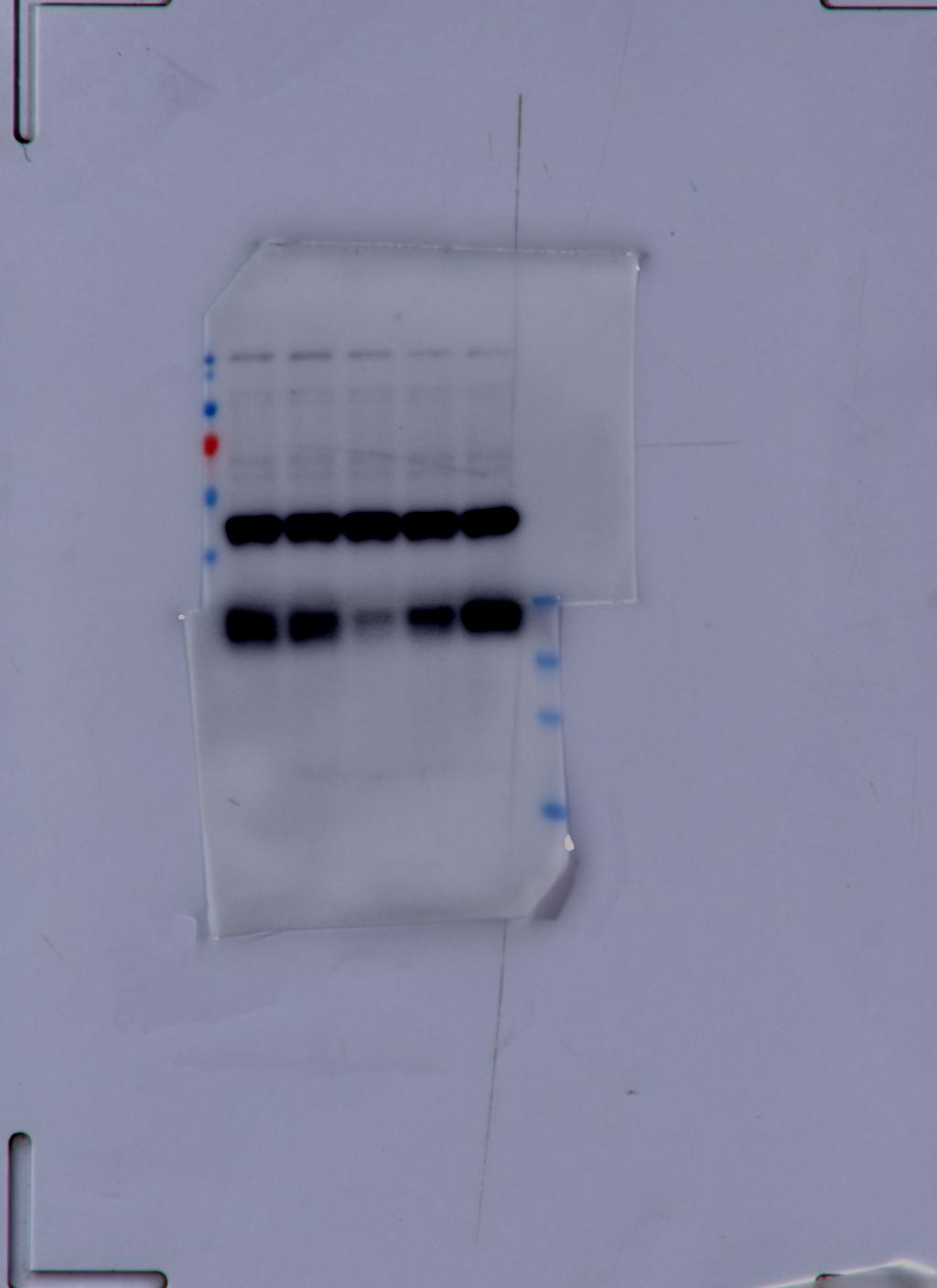

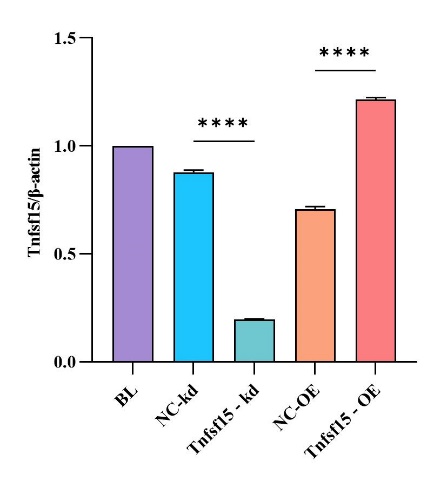


A.

B.

Tnfsf15

β-actin

BL

NC-kd

Tnfsf15-kd

NC-OE

Tnfsf15-OE

C.

D.

Supplement: Supplementary file 3 — Additional file 3. Verification of Tnfsf15 gene knockdown and overexpression. (A) Effect of RNA interference on the mRNA expression of the Tnfsf15 gene. NC: small interfering RNA negative control; Tnfsf15-kd: treatment group with small interfering RNA targeting the Tnfsf15 gene. *** P < 0.001; Student’s t test. (B) Effects of overexpression on the mRNA expression of the Tnfsf15 gene. NC: negative control group; Tnfsf15-OE: treatment group with the Tnfsf15 gene overexpression vector. The log2FC represents the log2-fold change. **P < 0.01; Student’s t test. (C) Verification of the effects of the RNA interference and overexpression plasmids on Tnfsf15 protein expression. (D) Band gray value ratios of the western blot data, with all the results normalized to those of β-actin. [file 13567_2025_1567_MOESM3_ESM.docx]
